# Supplementary material for: Protocol for a scoping review on information and communication technologies (ICTs) in community practice
Source: PLoS One. 2026 Apr 10;21(4):e0346869. doi: 10.1371/journal.pone.0346869 (PMC13068256; doi:10.1371/journal.pone.0346869)
Supplement: S1 Appendix — (DOCX) [file pone.0346869.s001.docx]

# Supporting Information

# Appendix S1: Search strategy for Sociological Abstracts [ProQuest].

| # | Searches |
| --- | --- |
| 1 | TIAB(“macro social work*” OR “collective action” OR “citizen participation” OR “civic participation” OR “public advocacy” OR “community participation” OR “grassroots coalition*” OR “grassroots activis*” OR “grassroots advocacy” OR “coalition building” OR “grassroots organiz*” OR “grassroots organis*” OR “community development” OR “adult education” OR “popular education” OR “capacity building” OR (community NEAR/2 organiz*) OR (community NEAR/2 organis*) OR ((community NEAR/2 practice) NOT “community of practice”) OR “community practitioner*” OR “community work*” OR (community NEAR/2 action) OR (community NEAR/2 activis*) OR (community NEAR/2 advocacy*) OR (community NEAR/2 “needs assessment”) OR “community building” OR “community engagement*” OR (community NEAR/2 intervention*) OR (community NEAR/2 leader*) OR (community NEAR/2 mobiliz*) OR (community NEAR/2 sensitiz*) OR (community NEAR/2 outreach) OR (community NEAR/2 planning) OR (community NEAR/2 “policy making”) OR (community NEAR/2 policymaking) OR (community NEAR/2 “program development”) OR “community social work*” OR (neighbo?r* NEAR/2 action) OR (neighbo?r* NEAR/2 organis*) OR (neighbo?r* NEAR/2 organiz*) OR (neighbo?r* NEAR/2 mobiliz*) OR (participatory NEAR/2 advocacy) OR (participatory NEAR/2 planning) OR (participatory NEAR/2 “policy making”) OR (participatory NEAR/2 policymaking) OR (participatory NEAR/2 “program development”)) |
| 2 | MAINSUBJECT.EXACT("Community organizations") OR MAINSUBJECT.EXACT("Radical pedagogy") OR MAINSUBJECT.EXACT("Community development") OR MAINSUBJECT.EXACT.EXPLODE("Advocacy") OR MAINSUBJECT.EXACT("Local planning") OR MAINSUBJECT.EXACT.EXPLODE("Citizen participation") OR MAINSUBJECT.EXACT("Collective action") OR MAINSUBJECT.EXACT("Neighborhood change") OR MAINSUBJECT.EXACT.EXPLODE("Community involvement") OR MAINSUBJECT.EXACT.EXPLODE("Mobilization") OR MAINSUBJECT.EXACT("Adult education") |
| 3 | 1 OR 2 |
| 4 | TIAB(algorithm* OR AI OR “artificial intelligence” OR “messaging app” OR messenger OR “mobile app*” OR (data NEAR/2 activism) OR (data NEAR/2 sovereignty) OR “datafication” OR “big data” OR blog* OR computer* OR cyber OR digital* OR media OR electronic OR email* OR facebook OR “geographic information system” OR GIS OR “global positioning system” OR GPS OR hardware OR ICT OR ICTs OR “information communication technolog*” OR “information technolog*” OR “information system*” OR instagram OR internet OR linkedin OR mobile* OR “social media” OR “online communication” OR smartphone* OR cellphone* OR phone* OR podcast* OR photovoice OR robot* OR remote OR skype OR software OR technolog* OR telework* OR teleconference* OR telecommunication* OR video* OR telegram OR tiktok OR tumbler OR twitter OR tweet* OR “augmented reality” OR “mixed reality” OR “extended reality” OR virtual OR wearable OR website* OR webcam* OR webinar* OR whatsapp OR youtube OR zoom OR chat* OR sms OR stream* OR tv OR telephone* OR television* OR texting OR radio*) |
| 5 | MAINSUBJECT.EXACT.EXPLODE(“Text messaging”) OR MAINSUBJECT.EXACT.EXPLODE(“Digital media”) OR MAINSUBJECT.EXACT.EXPLODE(“Big Data”) OR MAINSUBJECT.EXACT.EXPLODE(“Algorithms”) OR MAINSUBJECT.EXACT.EXPLODE(“Mobile phones”) OR MAINSUBJECT.EXACT.EXPLODE(“Artificial intelligence”) OR MAINSUBJECT.EXACT.EXPLODE(“Computer mediated communication”) OR MAINSUBJECT.EXACT.EXPLODE(“Social media”) OR MAINSUBJECT.EXACT.EXPLODE(“Internet”) OR MAINSUBJECT.EXACT.EXPLODE(“Computers”) OR MAINSUBJECT.EXACT.EXPLODE(“Telecommunications”) OR MAINSUBJECT.EXACT.EXPLODE(“Computer software”) OR MAINSUBJECT.EXACT.EXPLODE(“Human-computer interaction”) OR MAINSUBJECT.EXACT("Internet access") OR MAINSUBJECT.EXACT.EXPLODE(“Blogs”) OR MAINSUBJECT.EXACT.EXPLODE(“Geographic information systems”) OR MAINSUBJECT.EXACT.EXPLODE(“Electronic technology”) OR MAINSUBJECT.EXACT.EXPLODE(“Information technology”) OR MAINSUBJECT.EXACT.EXPLODE(“Human technology relationship”) OR MAINSUBJECT.EXACT.EXPLODE(“Virtual reality”) OR MAINSUBJECT.EXACT.EXPLODE(“Websites”) |
| 6 | 4 OR 5 |
| 7 | 3 AND 6 |
| 8 | 7 AND lan.exact(“ENG”) |
| 9 | 8 AND pd(20150101––) |
|  | DATE OF SEARCH: |
