# Supplementary material for: Protocol for a scoping review on information and communication technologies (ICTs) in community practice
Source: PLoS One. 2026 Apr 10;21(4):e0346869. doi: 10.1371/journal.pone.0346869 (PMC13068256; doi:10.1371/journal.pone.0346869)
Supplement: Attachment — Submitted filename: Response to Reviewers 23 March 2026.docx [file pone.0346869.s004.docx]

March 24, 2026

RE: Response for Reviewers for a Study Protocol: ***“Protocol for a Scoping Review on Information and Communication Technologies (ICTs) in Community Practice”***

Dear Academic Editor Wei Lang and the *PLOS One* Editorial Board,

On behalf of my co-authors, PhD Candidate Ali Pearson and Professor Stephanie Begun, I am submitting the enclosed revised Study Protocol for review by the *PLOS One* editorial Board. We thank you for your thorough revisions and are confident that your comments will improve the final manuscript.

**Reviewer #1:**

The manuscript is methodologically rigorous and fills a critical gap in the intersection of social work and ICTs research, holding significant theoretical and practical value. To further enhance its academic impact, improvements could be made in the following areas:

***Methodology***

(1) Search Strategy: The manuscript adheres to the Arksey & O’Malley framework and PRISMA-P guidelines, demonstrating a robust methodological foundation. But also needs to include concrete examples of search strings (e.g., for Sociological Abstracts) and explain optimizations (e.g., synonym expansion).
    **Response**: Thank you for this comment. We elaborated our search strategy optimization methods (e.g., thesaurus and keyword search, truncations, wildcards) under the subtitle “Identifying Relevant Literature” in the Methods section. An example of a complete search string is attached, as supporting information (S1). The string is too long to be included in the manuscript itself.
(2) Geographical Restrictions: The rationale for limiting empirical studies to the U.S. and Canada requires stronger justification. Clarify how these regions’ community practice theories and practices align theoretically to avoid exclusionary biases.

**Response**: Thank you for this comment. We provided a stronger justification for limiting empirical to the U.S. and Canada under “Screening Eligible Studies” in the Methods section. We also address the potential limitations posed by these geographical restrictions in our Discussion section.

(3) Temporal Scope:
    The 2015–2025 timeframe risks omitting pre-pandemic or foundational studies. Address how this choice might influence the comprehensiveness of findings.

**Response**: We mentioned the pandemic as a reference point to justify the 2015–2025 timeframe to demonstrate that this timeframe can also be productive in tracing the impact of COVID-19 on the relationships between ICTs and community practice. While findings may suggest insights into the impact of the pandemic on the relationship between community practice and ICTs, this is not the primary question asked in this review. Accordingly, we provided a more detailed rationale for this timeframe, anchoring it in relation to the Social Work Grand Challenges framework (specifically Harness Technology for Social Good).

***Data analysis***

(1) Quantitative Analysis:
    Specify hypothesis testing methods (e.g., chi-square tests, t-tests) for bivariate analyses to strengthen rigor.

**Response**: We elaborated on hypothesis testing methods in Data Analysis and Synthesis in the Methods section.

(2) Qualitative Analysis:
    Elaborate on thematic analysis procedures, including coding processes, theme generation methods, and intercoder reliability checks.
    **Response**: We elaborated on thematic analysis procedures in Data Analysis and Synthesis in the Methods section.

**Additional Changes #:**

(1) **Refining Conceptualization**: Based on the community social work literature, we replaced the expression “grassroots organizing/advocacy” with “community organizing”. In the North American social work literature, the term “community organizing” is widely used to encompass local grassroots organizing, organizational coalition building, and participatory advocacy.
(2) **Proofreading & Clarifications**: We thoroughly proofread and added clarification in several places throughout the manuscript. All changes can be traced in the track-changes version of the manuscript.

Sincerely,

Ofir Sivan, MSW, RSW

PhD Student, Factor-Inwentash Faculty of Social Work, University of Toronto, Ontario, Canada
